# Flexural Behavior and Fracture Mechanisms of Short Carbon Fiber Reinforced Polyether-Ether-Ketone Composites at Various Ambient Temperatures

**DOI:** 10.3390/polym11010018

**Published:** 2018-12-23

**Authors:** Bing Zheng, Tianzhengxiong Deng, Maoyuan Li, Zhigao Huang, Huamin Zhou, Dequn Li

**Affiliations:** State Key Laboratory of Material Processing and Die & Mold Technology, Huazhong University of Science and Technology, Wuhan 430074, China; zhengbing@hust.edu.cn (B.Z.); uezuuezu@hust.edu.cn (T.D.); limaoyuan@hust.edu.cn (M.L.); hmzhou@hust.edu.cn (H.Z.); ldq@hust.edu.cn (D.L.)

**Keywords:** SCF, PEEK, thermal properties, flexural behavior, microstructure, fracture mechanisms

## Abstract

In this study, the flexural behavior and fracture mechanisms of short carbon fiber reinforced polyether-ether-ketone (SCFR/PEEK) composites at various ambient temperatures were investigated. First, the crystallinity and glass transition temperature (*T*_g_) of PEEK and SCFR/PEEK were analyzed by differential scanning calorimetry analysis and dynamic mechanical analysis tests, respectively. The addition of SCFs increases the *T*_g_ but does not change the crystallinity of the PEEK matrix. Then, the three-point flexural tests of PEEK and SCFR/PEEK were performed over the temperature range of 20 to 235 °C, and the temperature-dependencies of the flexural properties of PEEK and SCFR/PEEK were discussed in detail. Finally, the microstructure of SCFR/PEEK was observed using a digital microscope and scanning electron microscope. The results show that the tension crack occurs first, and the crack extends upward leading to the shear crack and compression crack at room temperature. The fracture of SCFR/PEEK is mainly due to the extraction and rupture of SCFs. At high temperatures (above *T*_g_), the tension crack and compression crack both occur, and the strong ductility of the matrix prevents the generation of shear crack. The fracture of SCFR/PEEK is mainly due to the rotation and extraction of SCFs, while the SCFs rupture plays a minor role.

## 1. Introduction

Poly-ether-ether-ketone (PEEK), a unique high performance semi-crystalline polymer, is one of the most potential thermoplastics for medicine, automotive and aerospace applications, with the advantage of good impact performance, chemical inertness, heat resistance and biocompatibility [1,2,3,4]. These applications require thermal stability at elevated temperatures, and PEEK is one of the few polymers that can provide thermal durability and relatively high performance at elevated temperatures compared to metals [5]. However, pure PEEK is not appropriate for structural applications due to its relatively low stiffness and strength [6]. Short carbon fiber reinforced PEEK (SCFR/PEEK) is a moderately reinforced system between PEEK and continuous fiber reinforced PEEK, which significantly improves the stiffness and strength [7]. Since SCFR/PEEK contains the discontinuous SCFs, they cannot compete in stiffness and strength with the classical unidirectional long fiber composites. This disadvantage, however, also greatly reduces the difficulty of processing for SCFR/PEEK, resulting in the adoption of economical manufacturing methods such as extrusion and injection molding permitting wide practical applications [8,9].

In the past few decades, many efforts had been taken to investigate the mechanical properties of SCFR/PEEK [6,8,10,11,12]. Sarasua et al. [8] studied the mechanical behavior of short glass reinforced PEEK (SGFR-PEEK) and SCFR-PEEK composites, and also compared the interfacial shear strength and critical fiber length at break of two composites using the unidirectional tensile test and immersion ultrasonic technique at room temperature. Chi-Cherng Jeng et al. [10] investigated the effect of trans-crystalline interphase on the flexural properties of two kinds of SCFR/PEEK composites at room temperature, and the flexural failure mechanisms were discussed. They proved that the trans-crystalline interphase enhances the fiber/matrix adhesion, and improves the flexural deflection and strength of the composite. Moreover, they explained that compressive cracks occurred first and the tension crack then initiated from the tension side, met with the lower shear crack near the core region, and resulted in catastrophic failure. Sınmazcelik et al. [11] studied the effects of thermal aging on the bending and tribological properties of unfilled and random oriented SCRF/PEEK composites. Thermal aging increases the flexural properties of materials, and the tribological properties can be improved by enhancing mechanical properties of polymers and reducing adhesion in the contact area. Steinberg et al. [12] studied the biomechanical properties of SCFR/PEEK composites by four-point bending, static torsion and bending fatigue tests, for use as orthopedic implants including similar modulus to bone and ability to withstand prolonged fatigue strain. The results show that the Piccolo CF-PEEK Optima devices provided the recommended requirements for strength and wear for new devices and were safe and effective for intended use in humans. Chen et al. [6] investigated the uniaxial tensile properties of PEEK, SCFR/PEEK and SGFR/PEEK composites at various strain rates range from 10^−3^ to 10^3^ s^−1^ at room temperature. The paper shows the rate dependency on mechanical properties of SFR/PEEK, and the relationship between failure behavior with strain rate and fiber type. 

Although many experiments have been conducted, most efforts focused on the mechanical properties of SCFR/PEEK composite at room temperature, and few researchers investigated the flexural behavior and fracture mechanisms of SCFR/PEEK at various temperatures. Considering the durability and structural integrity of the materials are strongly affected by the service environments like high and low temperature [13], investigation of temperature-dependency on the flexural properties and fracture mechanisms of SCRF/PEEK composites is necessary. Flexural tests are widely used for mechanical evaluation of materials such as metals and alloys for engine turbines [14], mostly for ceramics and composites in aerospace applications [15,16,17,18,19,20,21], and even for medical implantation [9,12]. From the curves of the flexural response, the flexural elastic modulus, flexural strength and the fracture toughness, which are of great significance in engineering design and application, can be obtained [14]. 

The present study aims to further investigate the temperature-dependency on the flexural properties of SCFR/PEEK composites, and systematically discuss the fracture mechanisms at low and high temperature. First, the crystallinity and glass transition temperature (*T*_g_) of PEEK and SCFR/PEEK were analyzed by differential scanning calorimetry (DSC) analysis and dynamic mechanical analysis (DMA) tests, respectively. Then, the temperature-dependency on the flexural behaviors of PEEK and SCFR/PEEK were analyzed using three-point flexural tests over a wide range of temperatures from 20 to 235 °C. Furthermore, the microstructure of SCFR/PEEK was observed using a digital microscope and a scanning electron microscope (SEM). Finally, the flexural fracture mechanisms of SCFR/PEEK at low and high temperature were systematically discussed.

## 2. Materials and Methods 

### 2.1. Materials

In this study, the PEEK 450 and PEEK 450CA30 were supplied by Victrex ® High-performance Materials (Shanghai) Co, Ltd. (Shanghai, China), which were obtained by injection molding and the processing conditions are shown in Table 1. The weight content of SCF in the composites was 30%, and the diameter and average lengths of fibers were 10 and 24 μm, respectively. The melting temperature (*T*_m_) of PEEK was between 343 to 383 °C, and the glass transition temperature (*T*_g_) was about 143 to 163 °C. The density of PEEK and carbon fiber were 1300 kg/m ^3^ and 1760 kg/m ^3^, respectively. 

### 2.2. Thermal Analysis 

DSC analysis was performed in a differential scanning calorimeter (Diamond DSC, PerkinElmer Instruments, Shanghai, China) to determine the *T*_g_, *T*_m_, heat fusion (*H*_m_) and degree of crystallinity (*X*_c_) of the PEEK and SCFR/PEEK. This test was performed over the temperature range of 50 to 380 °C, with a heating rate of 10 °C/min, under a nitrogen atmosphere. The degree of crystallinity was determined using the following Equation [22]:

(1) $$X_{c}=\frac{\Delta H_{m}}{H_{f}(1-\alpha )}\times 100\%,$$

where, $X_{c}$ was the degree of crystallinity; $\Delta H_{m}$ was the enthalpy of fusion measured at the melting point, *T*_m_; $H_{f}$ was the enthalpy of fusion of the completely crystalline polymer, measured at the equilibrium melting temperature; $H_{f}$ was 130 J/g [23], α was the weight content of SCFs in the composites.

DMA tests were performed in a dynamic thermomechanical analysis machine (Diamond DMA, PerkinElmer Instruments, Shanghai, China) to investigate the glass transition temperature and dynamic mechanical of PEEK and SCFR/PEEK at various temperatures. Rectangular specimens with approximate dimensions of 40 mm × 5 mm × 2 mm were tested in three-point flexural mode, and the span was 20 mm. The strain history was a sinusoid with a peak amplitude of 0.01%, and the temperature was swept from 20 to 235 °C at a heating rate of 3 °C/min with frequencies of 1 Hz. The material was subjected to cyclic stress during the DMA test, and the viscoelastic properties of the material could be characterized by the stress response and strain response recorded in the experimental process. The relationship between storage modulus (*E*′), loss modulus (*E*″), loss tangent (tan δ) and temperature can be directly measured from the DMA test. The *E*′ was a representation of the elastic modulus of the material, while the *E*″ reflected the viscous modulus. The corresponding loss tangent (tan δ) was measured to confirm the *T*_g_ of PEEK and SCFR/PEEK.

### 2.3. Three-Point Flexural Tests

**Figure 1:**
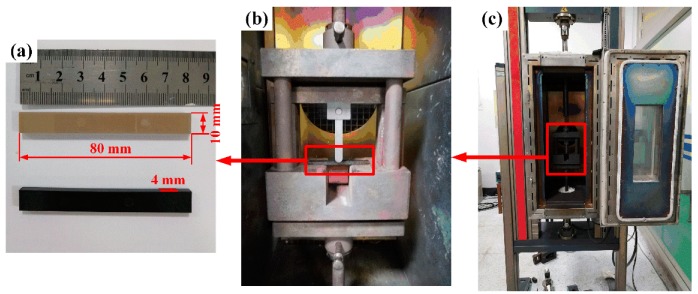
Representation of thermomechanical quasi-static flexural test: (**a**) Flexural specimens; (**b**) Flexural mold; (**c**) Test equipment.

Quasi-static three-point flexural tests were performed using an electrical universal testing machine (Z020, Zwick/Roell) at a constant speed of 2 mm/min, according to ISO 178. Rectangular specimens with approximate dimensions of 80 mm × 10 mm × 4 mm were tested, and the span was 40 mm. The flexural behavior of PEEK and SCRF/PEEK were investigated over a wide range of temperatures from 20 to 235 °C. The specimens were kept at room temperature for three days before testing, and three samples were tested for each experimental condition. Figure 1a shows the dimensions of flexural specimens according to ISO 178. Figure 1b,c show the mold and test equipment. 

In this study, the force-deflection curves were obtained. The flexural stress *σ*_b_, flexural strain *ε*_b_ and flexural modulus *E*_b_ were calculated as follows:

(2) $$\sigma_{b}=\frac{3FL}{2bh^{2}},$$

(3) $$\varepsilon_{b}=\frac{6sh}{L^{2}},$$

(4) $$E_{b}=\frac{\sigma_{b2}-\sigma_{b1}}{\varepsilon_{b2}-\varepsilon_{b1}},$$

where *F* and *s* were the force and deflection, respectively. *B* and *h* were the width and thickness of the specimens, and *L* was the span of the test section.

### 2.4. Microstructure Observation 

The microstructures of the SCFR/PEEK were observed using the super depth of field three-dimensional digital microscope (VHX-1000C, KEYENCE, Osaka, Japan) and field emission scanning electron microscope (JSM-7600F, JEOL, Beijing, China). The failure samples, which were tested at 20, 125, 165 and 235 °C, were selected. The optical microscope samples were embedded in a transparent resin and polished to expose a fractured cross section for observation. The SEM samples were sprayed with gold for 300 s to enhance the conductivity of the fracture surface before the test. The fractography analysis of the failed specimens was proven to be sufficiently relevant to study the damage mechanisms [24].

## 3. Results and Discussions

### 3.1. Thermal Analysis

**Figure 2:**
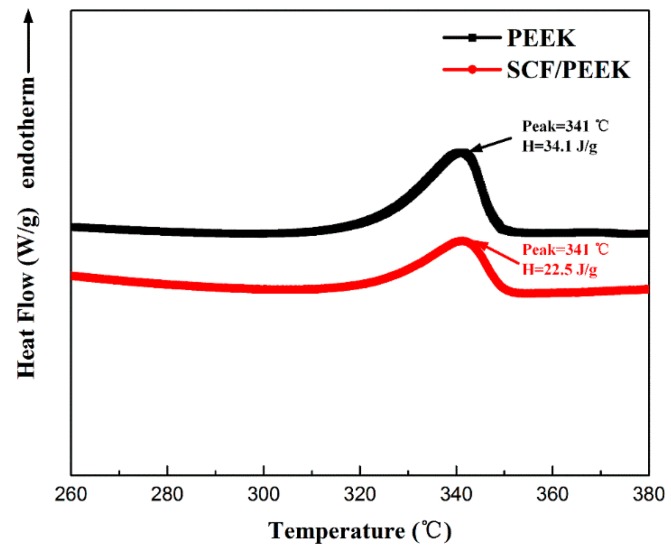
Differential scanning calorimetry (DSC) analysis of PEEK and short carbon fiber reinforced (SCFR)/PEEK composites.

**Figure 3:**
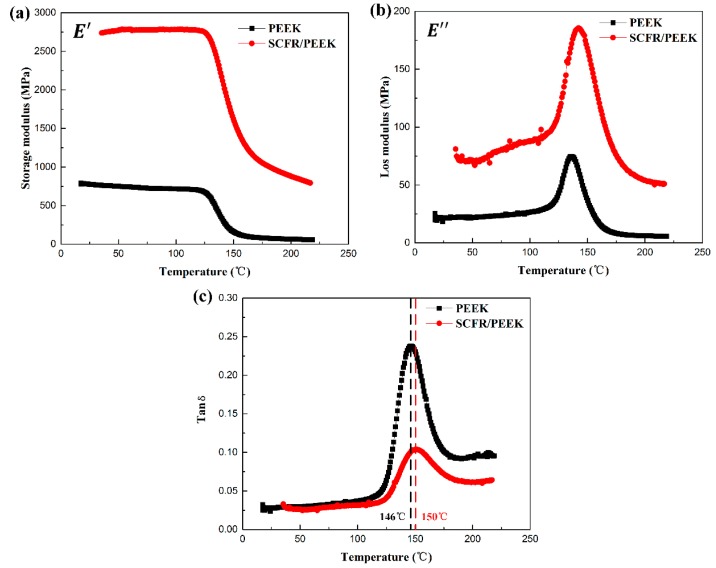
Dynamic mechanical analysis (DMA) results of PEEK and SCFR/PEEK composites: (**a**) the storage modulus ($E^{\prime }$) versus temperature; (**b**) the loss modulus ($E^{\prime \prime }$ ) versus temperature, (**c**) the tan δ versus temperature.

Figure 2 shows the DSC results of PEEK and SCFR/PEEK composites. It is known that the crystallization process significantly affects the properties of semi-crystalline polymers [25]. Therefore, it is important to analyze the influence of SCFs on the *T*_g_, *T*_m_, *H*_m_ and *X*_c_ of the PEEK matrix. As shown in Figure 2 and Table 2, the *T*_m_ and *X*_c_ of the PEEK and SCFR/PEEK remain relatively constant, with values of approximately 341 °C and 25 %, respectively. This shows that SCFs have not affected on the crystallization of the matrix, which is due to the annealing caused by the high mold temperature [26]. The values of Hm decrease from 34.1 to 22.5 J/g when the PEEK filled with SCFs, this is because that SCFR/PEEK has less matrix to release crystallization heat in the same volume. However, the values of *T*_g_ cannot be observed in the DSC curve. Thus, the *T*_g_ was obtained using the DMA analysis, and the addition of 30 % SCFs results in a higher value from 146 to 150 °C, as shown in Figure 3c.

Figure 3 is the DMA results of PEEK and SCFR/PEEK composites. The *E*′, *E*″ and tan δ versus temperature curves were presented in Figure 3a–c, respectively. As shown in Figure 3a, the *E*′ for SCFR/PEEK composite is higher than that of PEEK. The SCFs addition into the polymer matrix allows greater stress transfer at the interface, and the increase in *E*′ is mainly due to the stress transfer between the matrix and fibers [27]. Besides, the *E*′ of PEEK decreases gradually while that of SCFR/PEEK basically keeps consistent as the temperature increases from 20 to 125 °C. Then, there is a significant decrease in the *E*′ both of PEEK and SCFR/PEEK with the temperature increase from 125 to 150 °C. As the temperature rises, the rate of decrement of *E*′ with temperature decreases gradually. 

In Figure 3b, it can be seen that the *E*″ of PEEK and SCFR/PEEK both tend to improve initially and decline subsequently as the temperature is increased. The *E*″ indicates the viscous properties of the polymer, and generally, the viscosity of the materials decreases gradually as the temperature increases [28]. Before the peak, the material changes from a glassy state to a rubbery state, and the viscosity of the material increases as the temperature is increased. After the peak, the material is essentially in a rubbery state, and the viscosity of the material decreases as the temperature is increased. Moreover, the *E*″ for SCFR/PEEK composite is higher than that of PEEK, and the temperature of maximum heat dissipation of SCFR/PEEK is also larger than that of PEEK. The SCFs incorporated to matrix will enhance the resistance to viscous deformation and increase the *E*″. At the same time, the transition of the material from a glassy state to a rubbery state is more difficult due to the limitations of SCFs, resulting in the need for higher temperatures to reach peaks.

In Figure 3c, it can be seen that the tan δ value of PEEK and SCFR/PEEK both tend to improve initially and decline subsequently as the temperature is increased. The tan δ value of PEEK is higher than that of SCFR/PEEK. When the polymer begins to undergo a glass transition, the tan δ value will reach the maximum [29], which means the *T*_g_ of SCFR/PEEK is higher than that of PEEK. It is mainly due to the movement of polymer chains in the amorphous region of SCFR/PEEK composite is restricted by the interactions between fiber/fiber and fiber/matrix [28,30].

### 3.2. Flexural Behavior at Various Temperature

**Figure 4:**
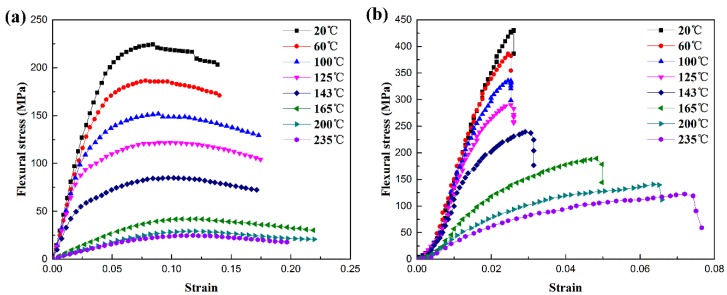
Flexural stress-strain curves of PEEK and SCFR/PEEK at various temperatures: (**a**) PEEK, (**b**) SCFR/PEEK.

**Figure 5:**
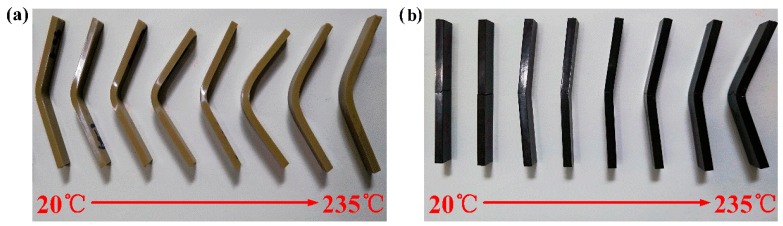
Flexural failure specimens of PEEK and SCFR/PEEK at various temperatures: (**a**) PEEK, (**b**) SCFR/PEEK.

Figure 4 and Figure 5 are the flexural curves and fracture specimens of PEEK and SCFR/PEEK at various temperatures, respectively. In Figure 4a, it can be observed that the flexural stress-strain curves of PEEK exhibit a typical elastic-viscoplastic behavior, which include three phases: elastic phase, overshoot phase typical of yield-stress fluids, and post-yielding phase showing a strain softening phenomenon. The yield phase and strain softening phase will increase while the elastic phase decreases when the temperature is increased. Moreover, the PEEK has strong flexural toughness and no damage occurs in the specimens at every test temperature, shown in Figure 5a. In Figure 4b, it can be observed that the flexural stress-strain curves of SCFR/PEEK exhibit an elastic behavior at room temperature, and the elastic phase will decrease and the viscoplastic behavior increase as the temperature increases. It is particularly necessary to note that there is a very small strain hardening phase at the initial stage. Then, they will break at the maximum point and the load drops off sharply when the fracture initiates. Besides, the brittle fracture of specimens is occurred at the low temperature, and the ductile fracture of specimens is observed as the temperature increases, shown in Figure 5b.

**Figure 6:**
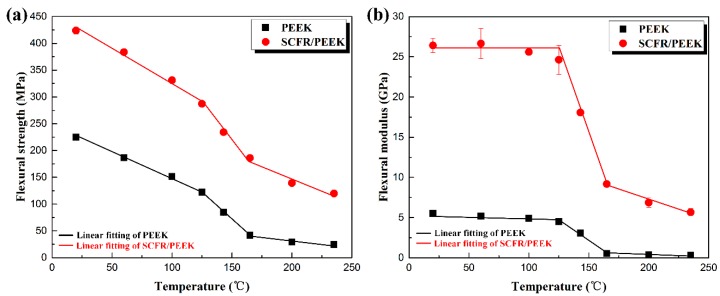
Relationship between flexural properties and temperature of PEEK and SCFR/PEEK: (**a**) Flexural strength vs. temperature; (**b**) Flexural modulus vs. temperature.

**Figure 7:**
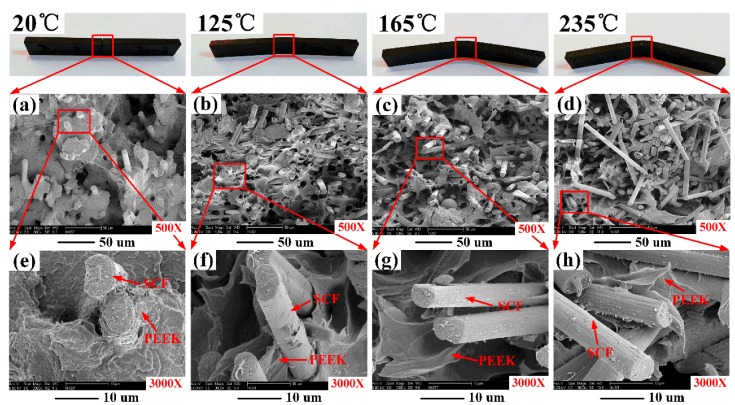
Scanning electron microscope (SEM) images of the fracture surfaces of SCFR/PEEK after flexural testing at various temperatures.

Figure 6 shows the relationship between flexural properties and temperature of PEEK and SCFR/PEEK. It is clearly evident that the flexural strength and modulus are strongly dependent on the testing temperature for both systems. In Figure 6, the flexural strength refers to the flexural stress at the maximum point on the stress-strain curve, and the flexural modulus is the ratio of flexural stress to flexural strain on the linear segment. The addition of SCFs effectively enhances the flexural strength and modulus, as shown in Figure 6. It is because of the interactions between the fiber/matrix and the obstruction of the SCFs to the crack expansion, which greatly enhance the performance of materials. In Figure 6a, it can be observed that the flexural strength of PEEK and SCFR/PEEK both tend to decline as the temperature increases, and the flexural modulus of PEEK and SCFR/PEEK tend to keep constant initially and decline subsequently as the temperature increases from 125 to 235 °C, as shown in Figure 6b, which is consistent with the trend shown in Figure 3a. In this temperature range, two competing mechanisms contribute to the behavior observed. First, the amorphous phase of polymer gradually changes from glassy state to rubbery state, the flexibility and free volume of polymer chain increase, which promotes the movement of the molecular chain [3,31]. Second, the bonding between the fiber and matrix is weakened as the temperature increases [32].These findings are supported by SEM images of the fracture samples, as shown in Figure 7.

Figure 7 shows the SEM images of the fracture surfaces of SCFR/PEEK after flexural testing at various temperatures. In Figure 7a, the PEEK is in the glassy state at the ambient temperature of 20 °C and the brittle fracture of PEEK matrix is observed. In general, the addition of SCFs leads to embrittlement of the ductile polymers [33,34]. Besides, a strong fiber/matrix interaction is observed according to the fracture surface, as shown in Figure 7e. In Figure 7b, the PEEK is partial softening at the ambient temperature of 125 °C, and the brittle fracture and ductile fracture are both observed in the fracture surface. Besides, the interaction between fiber/matrix becomes weaker, as shown in Figure 7f. When the ambient temperature is higher than the *T*_g_ (150 °C), the PEEK matrix had changed completely from glassy state to rubbery state, and the ductile fracture of the matrix is clearly observed in Figure 7c,d. Of course, the interaction between fiber-matrix is weak and there is no matrix coated on the pullout SCFs, as shown in Figure 7g,h.

### 3.3. Fracture Mechanisms Analysis

**Figure 8:**
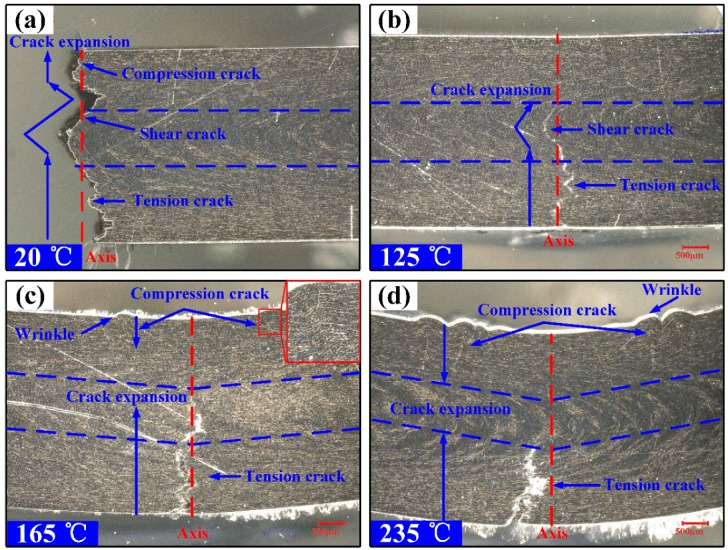
Micrographs of flexural failure specimens at various temperatures: (**a**) specimen failure at 20 °C, (**b**) specimen failure at 125 °C, (**c**) specimen failure at 165 °C, (**d**) specimen failure at 235 °C.

Figure 8 shows the micrographs of flexural failure specimens at various temperatures. In Figure 8a, it can be observed that the specimens tested at room temperature have brittle fractures. The tension crack, shear crack and compression crack occur in the lower surface, the core area and the upper surface, respectively. The crack expansion is instantaneous and catastrophic. In Figure 8b, the brittle and ductile are both observed in the fracture surface at the temperature of 125 °C. The brittle tension crack occurs in the upper surface and the shear crack occurs in the core area. However, the ductility of matrix caused by the increase of temperature prevents the further expansion of the crack, and there is no compression crack on the upper surface. In Figure 8c,d, it can be observed that the specimens tested above *T*_g_ are ductile fracture. The tension crack in the lower surface and compression crack in the upper surface both occurs, and there is no shear crack occurred in the core area. The tension crack will decrease but the compression crack increases as the ambient temperature is raised. Besides, the compression wrinkles and cracks occur on both sides of the axis.

**Figure 9:**
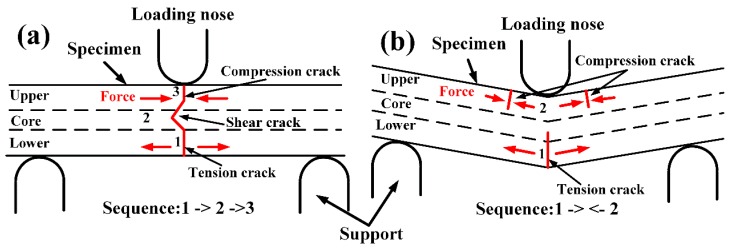
Schematic drawing of the fracture sequence of SCFR/PEEK composites at room temperature and high temperature: (**a**) specimen fracture at room temperature, (**b**) specimen fracture at high temperature, above *T*_g_.

In the three-point flexural test, there are three different crack types in the interior of the specimens: compression crack in the upper surface, tension crack in the lower surface and shear crack in the core area [10], as shown in Figure 8 and Figure 9. At room temperature, according the Figure 8a,b, it can be speculated that the tension crack (#1) in the lower surface occurs first. With further loading, the crack extends upward, with the result that the shear crack (#2) occurs in the core area and compression crack (#3) occurs in the upper surface, as shown in Figure 9a. This is mainly because the compression failure strength of SCFR/PEEK composites is higher than the tension failure strength [1]. This conclusion is inconsistent with the conclusion of reference [10]. They explained that compressive cracks occurred first and the tension crack then initiated from the tension side, met with the lower shear crack near the core region, and resulted in catastrophic failure. In addition, the compression cracks away from the contact zone are almost vertical crack along the axis. At high temperature (above *T*_g_), according the Figure 8c,d, the tension crack (#1) in the lower surface and the compression crack (#2) in the upper surface both occurs, and the strong ductility of matrix at the high temperature prevents the generation of shear cracks, as shown in Figure 9b. Besides, the compression cracks occur on both sides of the axis and perpendicular to the upper surface. Since the matrix is in a rubbery state and has a strong toughness, the SCFR/PEEK composites will destroy after a certain deflection, and the force exerted by the loading nose and supports meets near the axis, resulting in wrinkles and cracks.

**Figure 10:**
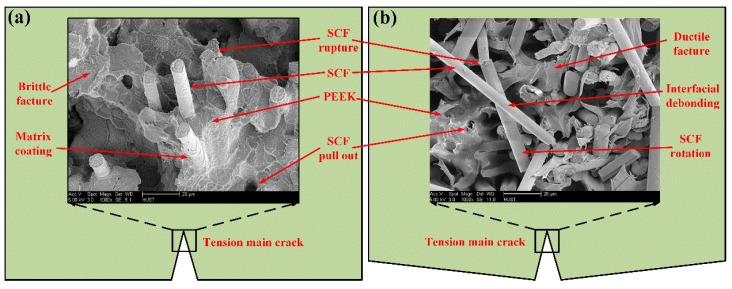
SEM of SCFR/PEEK composites depicting the flexural fracture mechanisms with the process zone ahead of the main tension crack at room temperature and high temperature: (**a**) room temperature, (**b**) high temperature, above *T*_g_.

Figure 10 is the SEM of SCFR/PEEK composites depicting the flexural fracture mechanisms with the process zone ahead of the main tension crack at room temperature and high temperature. In Figure 10a, it can be observed that the matrix is brittle fracture at room temperature. When the crack is expanding, the rotation of SCFs will be restricted by the brittle matrix and strong fiber/matrix interaction. Therefore, the fracture of SCFR/PEEK composites is mainly due to the rupture and extraction of SCFs. In Figure 10b, it can be observed that the matrix is ductile fracture at high temperature (above *T*_g_). The softening of matrix weakens the interaction between fibers and matrix, which makes the SCFs easy to rotate and pulled out when the crack is expanding. Therefore, the fracture of SCFR/PEEK composites is mainly due to the rotation and extraction of SCFs, while the SCFs rupture plays a minor role.

## 4. Conclusions

In this paper, the flexural behavior and fracture mechanisms of SCFR/PEEK composites at various ambient temperatures were investigated through thermal analysis, three-point flexural tests and microscopic observations.

(1) The thermal properties of PEEK and SCFR/PEEK were analyzed by DSC and DMA tests. The addition of 30% SCFs increases the *T*_g_ from 146 to 150 °C, but does not change the crystallinity of the PEEK matrix. Besides, the *E*′ and *E*″ for SCFR/PEEK composite are higher than that of PEEK, while the tan δ value for PEEK is higher than that of SCFR/PEEK. 

(2) The temperature-dependencies of the flexural properties of PEEK and SCFR/PEEK were performed by three-point flexural tests over the temperature range of 20 to 235 °C. The results show that the flexural stress-strain curves of PEEK exhibit a typical elastic-viscoplastic behavior and no damage occurs in the specimens at every temperature, while the flexural stress-strain curves of SCFR/PEEK exhibit an elastic behavior at room temperature and the viscoplastic behavior will increase when the temperature is increased. Moreover, the flexural strength of PEEK and SCFR/PEEK tend to decline while the flexural modulus tend to keep constant initially and decline subsequently as the temperature is increased. It is mainly due to the change of amorphous phase and the weakening of fiber/matrix interaction as the temperature increases. 

(3) The microstructure of SCFR/PEEK was observed by digital microscope and SEM. At room temperature, the tension crack occurs first in the lower surface, and the crack extends upward resulting in the shear crack and compression crack. The fracture of SCFR/PEEK is mainly due to extraction and rupture of SCFs. At high temperatures (above *T*_g_), the tension crack in the lower surface and compression cracks in the upper surface both occur, and the strong ductility of matrix prevents the generation of shear cracks. The tension crack will decrease but the compression crack increases as the ambient temperature increases. Besides, the compression wrinkles and cracks occur on both sides of the axis and perpendicular to the upper surface. The fracture of SCFR/PEEK composite is mainly due to the rotation and extraction of SCFs, while the SCFs rupture plays a minor role.

## Figures and Tables

**Table 1:** The injection molding process of poly-ether-ether-ketone (PEEK) 450 and PEEK 450CA30.

Sample	Nozzle Temperature (°C)	Mold Temperature (°C)
PEEK 450	375	180
PEEK 450CA30	395	180

**Table 2:** The thermal analysis results of PEEK and SCFR/PEEK composites.

Sample	*T*_g_^a^ (°C)	*T*_m_ (°C)	*H*_m_ (J/g)	*α* (%)	*X*_c_ (%)
PEEK	146	341	34.1	0	26
SCFR/PEEK	150	341	22.5	30	25

^a^*T*_g_ values were obtained from dynamic mechanical analysis (DMA) analysis.
